# Digital Twins for 3D Confocal Microscopy: Near-Field, Far-Field, and Comparison with Experiments

**DOI:** 10.3390/s25072001

**Published:** 2025-03-22

**Authors:** Poul-Erik Hansen, Tobias Pahl, Liwei Fu, Ida Nielsen, Felix Rosenthal, Stephan Reichelt, Peter Lehmann, Astrid Tranum Rømer

**Affiliations:** 1 Danish Fundamental Metrology, Kogle Alle 5, DK-2970 Hørsholm, Denmark; 2 Measurement Technology Group, Faculty of Electrical Engineering and Computer Science, University of Kassel, Wilhelmshoeher Allee 71, 34121 Kassel, Germanyp.lehmann@uni-kassel.de (P.L.); 3 Institute of Applied Optics, University of Stuttgart, Pfaffenwaldring 9, 70569 Stuttgart, Germanystephan.reichelt@ito.uni-stuttgart.de (S.R.)

**Keywords:** digital twin, microscopy, confocal microscopy, metrology, near-field calculation, far-field calculation, FEM, FMM, BEM, numerical simulation

## Abstract

To push the boundaries of confocal microscopy beyond its current limitations by predicting sensor responses for complex surface geometries, we build digital twins using three rigorous models, the finite element method (FEM), Fourier modal method (FMM), and boundary element method (BEM) to model light–surface interactions. Fourier optics are then used to calculate the sensor signals at the back focal plane and at the detector. A 3D illumination model is applied to 2D periodic structures for FEM and FMM modelings and to 3D aperiodic structures for BEM modeling. The lateral and vertical scanning processes of the confocal microscope are achieved through focal-point shifts of the objective, using plane-wave illuminations with varying incident and azimuthal angles. This approach reduces the need for repeated, time-intensive rigorous simulations of the scattering process when a fine scanning is desired. Furthermore, we give an in-depth description of a novel confocal microscopy method using FMM. For rectangular grating surfaces, the three models yield identical, highly accurate results, as validated by measured results. Simulations of the instrument transfer function, tilted gratings, and gratings with edge rounding offer insights into some experimentally observed effects. This research therefore provides a promising approach for correcting systematic errors in confocal microscopy.

## 1. Introduction

Micro-structures find a wide range of applications in optics, diagnostics, food science, sensing, and process inspection monitoring. Specific applications include enhancing waveguide coupling, improving linear encoders, developing hyperspectral cameras, and printing color images. Imaging technologies like optical microscopy (OM), atomic force microscopy (AFM), and scanning electron microscopy (SEM) are the dominating quality assessment technologies. While AFM and SEM measurements are too slow for high-volume manufacturing—with SEM being inherently destructive—OM also faces challenges in measurement accuracy due to the wave nature of light and the resulting diffraction effects, which cause the measured optical profile to deviate from the true surface.

Scanning confocal microscopy offers several advantages over conventional bright-field OMs. With this technique, the depth of the measuring field is controlled, and the scattered light away from the focal plane is reduced. It further enables measurements of objects with large variations in the vertical direction by serial imaging and achieves a higher lateral resolution compared to conventional optical microscopy. The key to the confocal advantages is the pinhole that eliminates out-of-focus light from the object, therefore providing a spatial filter. In addition, the use of a laser or a narrow line-width LED is crucial in order to form a coherent imaging system. In contrast to a normal incoherent bright-field microscope, which is described by linear addition of intensities, a digital-twin confocal microscope describes a coherent imaging system, and the signal is described by linear addition of all complex electromagnetic fields. The phase information in the complex fields thus usually requires a rigorous treatment of the light–matter interaction and the light propagation through the microscope [1,2,3,4].

Rigorous modeling of conventional microscopy images, including three-dimensional (3D) illumination, has been published by Totzeck and colleagues [5,6] based on the rigorous coupled-wave analysis (RCWA), also referred to as FMM. Török et al. [7] present a rigorous model for confocal microscopes based on the finite difference time domain (FDTD) method, where the scattering of a focused light beam is simulated with lateral and axial scanning by repeating the rigorous simulation. Pahl et al. [8] implemented a model for confocal microscopy based on FEM, where the lateral and axial scanning is considered in the post-processing leading to a reduction of simulation time since the time-consuming rigorous simulation solely needs to be repeated for discrete angles of incidence.

In this report, we compare three models for confocal microscopy that were developed with a focus on slightly different optical setups. These models are based on three rigorous simulation methods: FEM, FMM, and BEM. Despite being developed independently at different institutions with varying emphases and modeling requirements, the models yield similar results under identical conditions.

The insights gained from a rigorous simulation of an optical microscope allow for a more detailed understanding of the light propagation and the measured signal. A digital-twin confocal microscope (CM) can enhance measurement precision by allowing controlled exploration of potential deviations in the optical profile. We demonstrate how digital twins may be used to obtain insight into optical profile deviations like tilts and corner roundings. Furthermore, to demonstrate the capability of our digital-twin models, this report includes the effects of edge roundings and substrate tilt, in contrast to our earlier work [9].

We give an in-depth model description of confocal microscopy using FMM, which has been introduced only briefly in [9]. This method is robust, fast, and straightforward to implement using existing FMM solvers, making it suitable for numerous applications. To the authors’ knowledge, it has not been done before. The FMM description includes back focal plane (BFP) and image plane confocal microscopy, lateral scanning of the focus point without repeating the calculation for each point, post-processing calculation of the back focal plane image to the image plane, vertical scanning of the focus position in the post-processing step, and speed enhancement using symmetry which leads to a large reduction in the simulation time and makes the method a candidate for simulation of 3D structures. The back focal plane ability of the method enlarge the application range to fields like ellipsometry, scatterometry, focus microscopy, and ptychography.

This article is organized in the following way. In Section 3, we give an overview of the working principle for the digital twins used in this study. In Section 4, we explain how the near field is calculated by the different methods before we continue with a comparison of the near fields in Section 5. Section 5 ends with a comparison of simulated optical profiles using different methods and a discussion concerning the effects of corner roundings, sample tilt, and instrument transfer function. The article is closed with the conclusion in Section 6, in which we discuss the findings of this article.

## 2. Experiment

**Figure 1:**
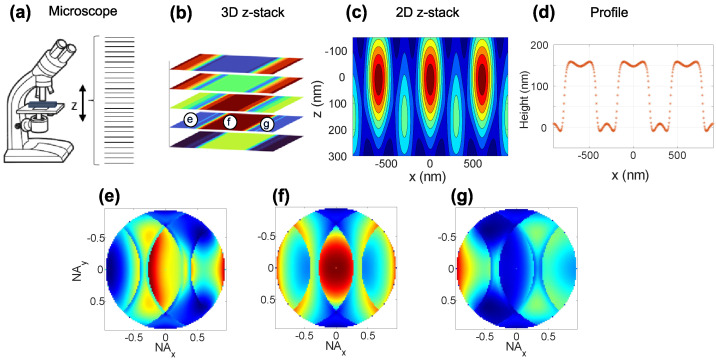
Figure (**a**–**d**) in the upper row illustrates the method for constructing the optical profile; (**a**) shows the vertical scanning of the optical microscope, (**b**) shows captured 2D images at selected vertical positions, (**c**) shows the height map obtained from vertical scanning of the microscope and (**d**) shows the optical profile calculated from the height map in (**c**). Figure (**e**–**g**) in the lower row shows the y-polarized back focal plane diffraction efficiency images corresponding to the circular points indicated in (**b**).

All the measurements are done with an Olympus Lext 5100 Laser scanning microscope (Olympus, Tokyo, Japan) using laser wavelength of 405 nm, an infinity-corrected objective with a numerical aperture (NA) of 0.95 and magnification of 100×. The vertical distance between two 2D images is set to 120 nm, and the lateral scan width is set to 16 μm by applying a zoom factor of 8 to the beam scanning galvanometers. Optical 2D images are acquired for each fixed vertical position as the sample is scanned horizontally with 1024 pixels in each lateral direction. Each vertical scan position results in slightly different two-dimensional images that are stacked at their corresponding scan position (x,y,z), creating a 3D image, where the *x* and the y-axes represent the lateral position on the target and the z-axis the scanned focus position, see Figure 1. The measurements were performed on nine SiMetricS RS-N silicon calibration (SiMETRICS, Chemnitz, Germany) gratings with nominal pitches from 300 nm to 6000 nm, nominal heights from 138 nm to 192 nm, and 50 % duty cycle [10].

## 3. Working Principles of the Digital Twins

**Figure 2:**
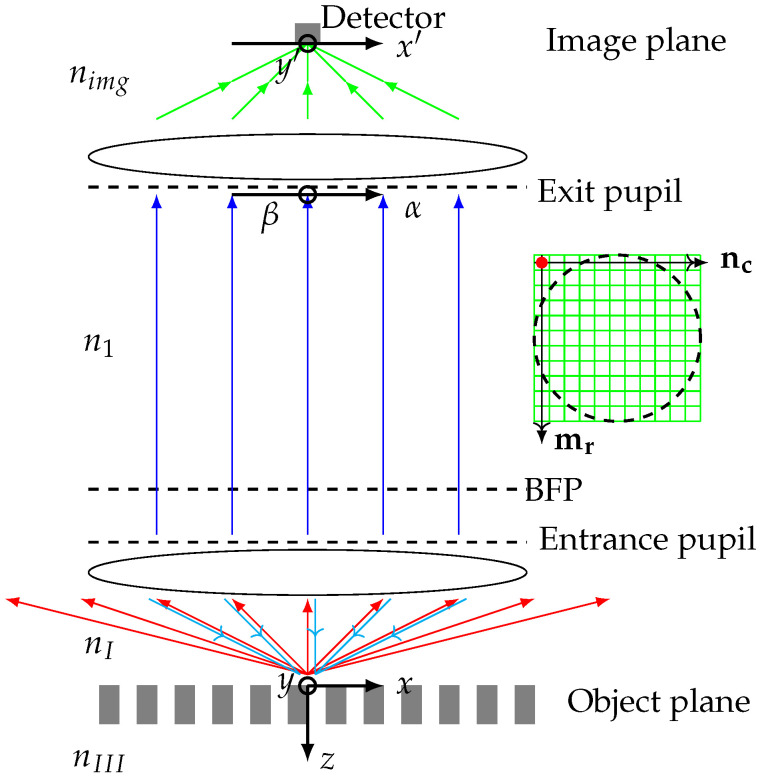
The vectorial image formation model for the digital-twin microscope. The cyan arrows illustrate the propagation of the incident field focused by the microscope objective. The red arrows illustrate the propagation of the scattered fields from the object. The blue arrows illustrate the field propagating between the entrance and the exit pupil. The green arrows illustrate the focusing of the field onto the detector. The inserted green square grid illustrates an exemplary meshing of the entrance and exit pupils, and the red dot shows that the mesh points are in the center of each mesh. The dashed line inside the mesh is the circumference of the microscope lenses. Only mesh points within the circumference are used in the simulations. Infinity-corrected microscopes are standard in the industry and have beam paths with parallel rays between the objective and the tube lens, as shown in this figure.

The working principle of a microscope is explained in terms of how the scattered light from the surface is collected and processed by the microscope. The processing is usually discussed at certain locations within the microscope called the object plane, the back focal plane and the image plane. The object plane is the light–surface interaction plane, the back focal plane is the location where the light field contains information on the angular spectrum that makes up the object, and the image plane is the plane where image formation takes place and where the detector is located. A visualization of the different planes and the light propagation is given in Figure 2. The back focal plane corresponds to the Fourier plane of the aplanatic lens and maybe the light-emitting plane and the detector plane for the far-field angular scattering of the light from the object [11,12,13]. The digital twin of a microscope works in the following way:

1. In the simulation, we define the light properties in a grid within the back focal plane. Each cell in the grid has an amplitude vector and a propagation vector. The amplitude vector is in the back focal plane and the propagation vector is perpendicular to the back focal plane, pointing towards the object plane. We say that the amplitude vector is x-polarized if the azimuth angle ϕ is zero with respect to the Cartesian x-axis in the back focal plane and y-polarized if ϕ is π/2. The periodic object structure is placed such that the periodicity is along the x-polarized direction in the back focal plane.
2. The light beam coordinate system defined by the light beam unit vectors for TE-amplitude, TM-amplitude, and the propagation vector is used to describe the optical propagation of the light within the microscope. Please note that x- and y- polarization refers to a Cartesian coordinate system in this study, whereas TE and TM polarization is defined with respect to the coordinate system of a certain light beam.
3. The response from the object structure is calculated using one of the three digital-twin scattering methods for a range of vertical distances between the microscope objective and the object, as well as for a range of lateral positions along the periodicity direction. Importantly, the simulations can be done for different lateral positions and vertical distances without repeating the time-consuming rigorous calculations of the light–matter interaction.
4. All the scattered light, for a given vertical and lateral position, that enters the same cell in the back focal plane grid is added coherently. At this point, the presented models differ in that the fields are added either in the back focal plane, as is the case of FMM, or in the image plane, as done in FEM and BEM. This discrepancy is caused by different working principles considered initially in the implementations. This, however, should not give rise to any deviations in the observables.
5. The 2D image formation is made by focusing the simulated far-field angular scattering in the back focal plane for a given vertical position onto the image plane. Each back focal image gives a point in the image plane; see Figure 1.
6. The 3D image stack is obtained by performing 2D image formations for all vertical positions and stacking the 2D images, as depicted in Figure 1a,b.
7. This process of profile extraction from a 3D image stack is given in two steps. First, we use the 2D nature of the grating object to reduce the 3D image stack to a 2D image stack, see Figure 1c. The vertical positions (*z*) are given on the vertical axis, and the lateral position (*x*) is on the horizontal axis. The colors show the signal intensity. For each lateral position, the optical profile is obtained by the vertical position, for which the intensity is maximal; see Figure 1d.

The optical lenses in the digital twins are at present assumed to be aplanatic, and the presented Fourier method relies on two rules: (1) *The sine condition* and (2) *the intensity law* [14].

Although the depth of field of a confocal microscope is of the order of hundreds of nanometers, the axial resolution is not. With respect to Figure 1c, the axial position of the maximum intensity, or the intensity signal’s centroid, for each pixel can be found with nanometer accuracy. Thus, samples with a height less than the depth of field can be reconstructed from measured intensities using conventional signal processing algorithms [15].

## 4. Introduction to the Scattering and Imaging Models

A detailed comparison of the three scattering models for plane waves is reported in Ref. [16]. Each of the three methods performs differently regarding computational effort, memory requirement, and versatility. However, with this study, we demonstrate that all models give similar results for scattered–focused field and microscope imaging, and we do not provide a list of advantages and disadvantages. For a general and more detailed overview of several rigorous methods, including the three presented here, we e.g., refer to Ref. [17].

### 4.1. FMM Method

Microscope imaging using FMM is a novel method for near-field and far-field imaging for confocal microscopy.

#### 4.1.1. Scattered Field for Focused Light

An intrinsic property of a lens is that it does not change the polarization state of linearly polarized light with polarization vectors in (TM) and perpendicular (TE) to the plane of incidence. To describe the polarization state, we choose unit vectors parallel and perpendicular to the plane of incidence and describe each plane of incidence by an angle ϕ relative to a fixed Cartesian coordinate system at the sample position. Hence, we introduce three light beam unit vectors $n_{TE}$, $n_{TM}^{cyl}$ and $n_{TM}^{sph}$. The vectors $n_{TE}$ and $n_{TM}^{cyl}$ are cylindrical unit vectors for the field amplitude in the back focal plane, where the light is described by an incoming plane wave of polarization parallel (TM) and perpendicular (TE) to the plane of incidence.

(1) $$\begin{array}{ccc} E_{TE}\left(r\right)=\underset{n_{TE}}{\underset{︸}{\left(\begin{array}{c} -\sin(\phi )\\ \cos(\phi )\\ 0 \end{array}\right)}}e^{ik\cdot r} & \mathrm{and} & E_{TM}^{cyl}\left(r\right)=\underset{n_{TM}^{cyl}}{\underset{︸}{\left(\begin{array}{c} \cos(\phi )\\ \sin(\phi )\\ 0 \end{array}\right)}}e^{ik\cdot r} \end{array}$$

To describe the light after the aplanatic lens, where the light is focused down, spherical coordinates are needed for the TM polarized light:

(2) $$\begin{array}{c} E_{TM}^{sph}\left(r\right)=\underset{n_{TM}^{sph}}{\underset{︸}{\left(\begin{array}{c} \cos(\theta )\cos(\phi )\\ \cos(\theta )\sin(\phi )\\ -\sin(\theta ) \end{array}\right)}}e^{ik\cdot r}. \end{array}$$

The amplitude (A) of an incident linearly polarized plane wave with polar angle θ=0 and an arbitrary azimuth angle, ϕ, is expressed as a sum of amplitudes along the *x*-axis ($A_{x}=A\cos\left(\phi \right)$) and the y-axis ($A_{y}=A\sin\left(\phi \right)$). This vector is projected onto the unit vectors $n_{TE}$ and $n_{TM}^{cyl}$

(3) $$\begin{array}{ccc} A_{TE}\left(\phi \right) & = & A\cdot n_{TE}=-A_{x}\left(\phi \right)\sin\left(\phi \right)+A_{y}\left(\phi \right)\cos\left(\phi \right)\; \end{array}$$

(4) $$\begin{array}{ccc} A_{TM}\left(\phi \right) & = & A\cdot n_{TM}^{cyl}=A_{x}\left(\phi \right)\cos\left(\phi \right)+A_{y}\left(\phi \right)\sin\left(\phi \right). \end{array}$$

The lens bends the incident field amplitude into a refracted field amplitude given by

(5) $$\begin{array}{ccc} A_{in}\left(\theta ,\phi \right) & = & A_{TE}\left(\phi \right)n_{TE}\left(\phi \right)+A_{TM}\left(\phi \right)n_{TM}^{Sph}\left(\theta ,\phi \right). \end{array}$$

The FMM boundary conditions for the calculation of the transmitted field ($E^{T}$), reflected field ($E^{R}$), and the field inside the grating layer ($E^{G}$) assume an incident amplitude with unit magnitude. In our case, the incident amplitudes are given by Equation (3), and we use the linearity of the problem to rewrite the standard expression in Ref. [18] for focused light onto a grating structure with period $\Lambda_{x}$. The Floquet conditions relate the incident propagation angle (θ,ϕ) to the outgoing/diffracted wave vector ${\left(k_{x,m}^{out},k_{y}^{out},k_{z,l,m}^{out}\right)}^{T}$ in the following way:

(6) $$\begin{array}{ccc} k_{x,m}^{out} & = & \left|k|(n_{I}\sin\left(\theta \right)\cos\left(\phi \right)-m\frac{\lambda }{\Lambda_{x}}\right)\\ k_{y}^{out} & = & \left|k\right|n_{I}\sin\left(\theta \right)\sin\left(\phi \right)\\ k_{z,l,m}^{out} & = & \left\{\begin{array}{cc} \sqrt{{\left|k\right|}^{2}n_{l}^{2}-{\left(k_{x,m}^{out}\right)}^{2}-{\left(k_{y}^{out}\right)}^{2}} & \left|k\right|n_{l}\geq \sqrt{{\left(k_{x,m}^{out}\right)}^{2}+{\left(k_{y}^{out}\right)}^{2}}\\ -j\sqrt{{\left(k_{x,m}^{out}\right)}^{2}+{\left(k_{y}^{out}\right)}^{2}-{\left|k\right|}^{2}n_{l}^{2}} & \left|k\right|n_{l}<\sqrt{{\left(k_{x,m}^{out}\right)}^{2}+{\left(k_{y}^{out}\right)}^{2}} \end{array}\right.\\ l & = & I,III \end{array}$$

where $n_{l}$, l=I,III denotes the complex refractive index in Region I, above the grating layer, and Region III, the substrate region of the grating layer, and λ is the wavelength. The grating layer is denoted by Region II. The transmitted field ($E^{T}$), reflected field ($E^{R}$), and the field inside the grating layer ($E^{G}$) for focused light may now be written as

(7) $$\begin{array}{ccc} \;E^{R} & = & A_{in}e^{-i\left(k_{x,m}x+k_{y}y+k_{z,I,m}z\right)}+A_{TE}\sum_{m}R_{m,TE}e^{-i\left(k_{x,m}x+k_{y}y-k_{z,I,m}z\right)}\\ & & +A_{TM}\sum_{m}R_{m,TM}e^{-i\left(k_{x,m}x+k_{y}y-k_{z,I,m}z\right)}\\ E^{T} & = & A_{TE}\sum_{m}T_{m,TE}e^{-i\left(k_{x,m}x+k_{y}y+k_{z,III,m}z\right)}+A_{TM}\sum_{m}T_{m,TM}e^{-i\left(k_{x,m}x+k_{y}y+k_{z,III,m}z\right)}\\ E^{G} & = & A_{TE}\sum_{m}S_{m,TE}\left(z\right)e^{-i\left(k_{x,m}x+k_{y}y\right)}+A_{TM}\sum_{m}S_{m,TM}\left(z\right)e^{-i\left(k_{x,m}x+k_{y}y\right)}. \end{array}$$

Here $R_{m}$, $S_{m}$ and $T_{m}$ are the electric field amplitudes of m’th order in Region I, II and III.We rewrite the Floquet conditions in order to obtain all possible incident wave vectors for a given outgoing k-vector. The outgoing k-vector is found by selecting a point $\left(n_{c},m_{r}\right)$ in the entrance pupil plane, Figure 2, and using Fourier optics for focusing the light. A microscope can only detect the propagating k-vectors that are caught by the microscope objective. This gives the following additional constraint for the diffracted wave vectors caught by the objective

(8) $$\begin{array}{c} \sqrt{{\left(k_{x,m}^{in}\right)}^{2}+{\left(k_{y}^{in}\right)}^{2}}\leq \left|k\right|\mathrm{NA} \end{array}$$

with incoming wave vectors given by $k_{x,m}^{in}=k_{x}^{out}+m\frac{\lambda }{\Lambda_{x}}$ and $k_{y}^{in}=k_{y}^{out}$. The selection of the incoming light as determined by Equation (8) is illustrated by the cyan arrows in Figure 2. The scattered–focused intensity in Region I, II, and III are found by integration over all valid incident angles $\alpha =k_{x}^{in}/\left|k\right|$ and $\beta =k_{y}^{in}/\left|k\right|$.

(9) $$\begin{array}{ccc} I^{R} & \propto & \underset{\sqrt{\alpha^{2}+\beta^{2}}\leq \mathrm{NA}}{\int \int }{\left|A_{TE}\sum_{m}R_{m,TE}e^{-i|k|\left(\alpha_{m}x+\beta y-\gamma_{I,m}z\right)}\right|}^{2}+{\left|A_{TM}\sum_{m}R_{m,TM}e^{-i|k|\left(\alpha_{m}x+\beta y-\gamma_{I,m}z\right)}\right|}^{2}d\alpha d\beta \\ I^{T} & \propto & \underset{\sqrt{\alpha^{2}+\beta^{2}}\leq \mathrm{NA}}{\int \int }{\left|A_{TE}\sum_{m}T_{m,TE}e^{-i|k|\left(\alpha_{m}x+\beta y+\gamma_{III,m}z\right)}\right|}^{2}+{\left|A_{TM}\sum_{m}T_{m,TM}e^{-i|k|\left(\alpha_{m}x+\beta y-\gamma_{III,m}z\right)}\right|}^{2}d\alpha d\beta \\ I^{G} & \propto & \underset{\sqrt{\alpha^{2}+\beta^{2}}\leq \mathrm{NA}}{\int \int }{\left|A_{TE}\sum_{m}S_{m,TE}\left(z\right)e^{-i|k|\left(\alpha_{m}x+\beta y\right)}\right|}^{2}+{\left|A_{TM}\sum_{m}S_{m,TM}\left(z\right)e^{-i|k|\left(\alpha_{m}x+\beta y\right)}\right|}^{2}d\alpha d\beta \end{array}$$

with $\gamma =k_{z}/\left|k\right|$.

#### 4.1.2. Propagation of Scattered Fields from the Object Plane to the Exit Pupil Plane

The field diffracted from the sample, as described in Section 4.1.1 is transmitted through the microscope objective and onto the exit pupil plane. For each selected position $\left(n_{c},m_{r}\right)$ in the entrance pupil plane, we find the outgoing k-vector from the focal point to the selected position. This outgoing k-vector direction is common for all contributing waves to the selected position, and we may use Equation (6) to find the contributing incident waves. When the object is placed in focus, the lens bends the outgoing wave such that the k-vectors, after the lens, are given by (0,0,|k|) and the TE and TM amplitudes are lying in the exit pupil plane. We obtain the outgoing amplitudes $A_{x,m}$ and $A_{y,m}$ from $A_{TE}R_{m,TE}$ and $A_{TM}R_{m,TM}$ by inverting Equation (3) and multiplying it by a propagation constant. This may be expressed mathematically as

(10) $$\begin{array}{c} \left[\begin{array}{c} A_{x,m}\\ A_{y,m} \end{array}\right]=\left(\sqrt{\frac{\cos(\theta )}{\cos(\theta_{s,m})}}\right)\left[\begin{array}{cc} \cos(\phi_{s,m}) & -\sin(\phi_{s,m})\\ \sin(\phi_{s,m}) & \cos(\phi_{s,m}) \end{array}\right]\left[\begin{array}{c} A_{TM}R_{TM,m}\\ A_{TE}R_{TE,m} \end{array}\right]. \end{array}$$

Here, θ and $\theta_{s,m}$ are the polar angles of the incident and outgoing fields, and $\phi_{s,m}$ is the scattered azimuth angle. All the exit pupil amplitudes, $A_{x,m}$ and $A_{y,m}$, with the same scattering angles $\theta_{s,m}$ and $\phi_{s,m}$, but different incident angles are added coherently before they are saved in the exit pupil amplitude vector $E_{ep}$. This is repeated for all valid mesh points in the exit pupil.

#### Symmetry Consideration

The back focal plane image exhibits mirror-plane symmetry in the (x,z) and (y,z) plane as shown in the center back focal image in Figure 1, if the incident illumination is homogeneous and the considered structure is symmetrical. We have developed and implemented an algorithm for FMM that makes full use of this symmetry to decrease the computational time. The algorithm first performs the calculation of all the reflected field amplitudes for the first quadrant (x>0 and y>0) and stores them. Secondly, the field amplitudes for the other quadrants are obtained by altering the sign and order of the stored field amplitudes to match the output from a direct calculation without symmetry. Finally, the remaining calculation points, x=0 and/or y=0, are obtained by direct calculation.

#### Lateral Scanning of Focus Position

The light–matter response from any lateral placement of the repeated structure within the unit cell is identical to within a phase factor. Mathematically this means that the light response for a new object location $\left(x,y_{0}\right)$ may be obtained from a known field at position $\left(x_{0},y_{0}\right)$ in the following way, for a repeated structure along the *x*-axis [19]

(11) $$\begin{array}{c} R_{m}\left(x,y_{0}\right)=R_{m}\left(x_{0},y_{0}\right)e^{i\frac{2\pi m(x-x_{0})}{\Lambda_{x}}}. \end{array}$$

Here m=−M,⋯,M is the number of Fourier modes along the *x*-axis and $\Lambda_{x}$ is the pitch along the *x*-axis.

#### Vertical Scanning of Focus Position

The light–matter interaction point is changed slightly during a through-focus height scan of the object. This shift is called the defocus distance, δz, and it is measured relative to the in-focus distance (z=0) in Figure 2. Following Refs. [8,20], this results in a phase shift given by

(12) $$\begin{array}{c} e^{i|k|\left(\gamma_{in}\left(\alpha_{in},\beta_{in}\right)+\gamma_{scat}\left(\alpha_{scat},\beta_{scat}\right)\right)\delta z}. \end{array}$$

Here |k| is the magnitude of the k-vector, and $\gamma_{(}\alpha_{in},\beta_{in})=\sqrt{1-\alpha_{in}^{2}-\beta_{in}^{2}}$ is the *z*-component of the normalized incident k-vector at location $\left(\alpha_{in},\beta_{in}\right)$ on the entrance pupil. Likewise $\gamma_{scat}$ is the *z*-component of the normalized scattered k-vector at location $\left(\alpha_{scat},\beta_{scat}\right)$ in the exit pupil, which for a given location $\left(\alpha_{scat},\beta_{scat}\right)$ is given by $\gamma_{scat}\left(\alpha_{scat},\beta_{scat}\right)=\sqrt{1-\alpha_{scat}^{2}-\beta_{scat}^{2}}$. The field amplitudes in Equation (10) for a given entrance pupil location, exit pupil location, and defocus distance δz now become

(13) $$\begin{array}{ccc} \;\left[\begin{array}{c} A_{x}\left(\alpha_{in},\beta_{in},\alpha_{scat},\beta_{scat},x,y_{0},\delta z\right)\\ A_{y}\left(\alpha_{in},\beta_{in},\alpha_{scat},\beta_{scat},x,y_{0},\delta z\right) \end{array}\right] & = & \left[\begin{array}{c} A_{x}\left(x,y_{0}\right)\\ A_{y}\left(x,y_{0}\right) \end{array}\right]\times \\ & & e^{i|k|\left(\gamma_{in}\left(\alpha_{in},\beta_{in}\right)+\gamma_{scat}\left(\alpha_{scat},\beta_{scat}\right)\right)\delta z}. \end{array}$$

The entrance and exit pupils are divided into the same number of cells/pixels in our implementation. This allows the use of (α,β) as the common notation for locations in the entrance and exit pupil. The phase in Equation (12) should be multiplied on each of the incident/scattered field components that contribute to the exit pupil field location (α,β). The field amplitude in an exit pupil mesh point (α,β) is written as $E_{ep}\left(\alpha ,\beta ,x,y_{0},\delta z\right)={\left(A_{x}\left(\alpha \right),A_{y0}\left(\beta \right)\right)}^{T}$. The exit pupil field is the incident field on the tube lens that focuses the light onto the image plane.

#### 4.1.3. Propagation of the Fields from the Exit Pupil Plane to the Image Plane

To determine the detector signal, we integrate over the exit pupil plane. This is carried out by a summation of the contributions from all the mesh points in the exit pupil. For a square mesh like ours, this is carried out most easily using normalized k-space coordinates α, β and γ. The connection to the azimuth angles of Equation (10) is given by $\sin\left(\phi \right)=\beta /\sqrt{\alpha^{2}+\beta^{2}}$ and $\cos\left(\phi \right)=\alpha /\sqrt{\alpha^{2}+\beta^{2}}$. An exit pupil mesh point has a (α,β) coordinate that can take values between −NA and NA with a uniform spacing dα in the α direction and dβ in the β direction. The area of a single mesh is therefore dαdβ. The additional constraint $\sqrt{\alpha^{2}+\beta^{2}}\leq \mathrm{NA}$ ensures that we only consider mesh points inside the exit pupil/tube lens, see Figure 2. The lens bends the incident exit pupil field amplitude $E_{ep}\left(\alpha ,\beta ,x,y_{0},\delta z\right)$ into a refracted field $E_{s}$ after the lens. The refracted field may be found by first projecting the incident field onto the TM and TE polarized unit vectors, $n_{TE}$ and $n_{TM}^{cyl}$, before the lens and secondly refract these two fields through the lens [14] according to

(14) $$\begin{array}{ccc} \;E_{s}\left(\alpha ,\beta ,\gamma^{\prime },x,y_{0},\delta z\right) & = & \left(t^{s}\left(\theta \right)\left[E_{ep}\left(\alpha ,\beta ,x,y_{0},\delta z\right)\cdot n_{TE}\right]n_{TE}\right.\\ & + & \left.t^{p}\left(\theta \right)\left[E_{ep}\left(\alpha ,\beta ,x,y_{0},\delta z\right)\cdot n_{TM}^{cyl}\right]n_{TM}^{sph}\right)\sqrt{\frac{n_{1}}{n_{img}}}\sqrt{\gamma^{\prime }}, \end{array}$$

where $n_{img}$ is the refractive index between the tube lens and the detector, and $t^{s}$ and $t^{p}$ are the Fresnel transmission coefficients. It is convenient to express the unit vectors in terms of normalized k-vectors in the following way:

(15) $$\begin{array}{ccc} n_{TE}=\left(\begin{array}{c} -\frac{\beta }{\sqrt{\alpha^{2}+\beta^{2}}}\\ \frac{\alpha }{\sqrt{\alpha^{2}+\beta^{2}}}\\ 0 \end{array}\right),n_{TM}^{cyl} & = & \left(\begin{array}{c} \frac{\alpha }{\sqrt{\alpha^{2}+\beta^{2}}}\\ \frac{\beta }{\sqrt{\alpha^{2}+\beta^{2}}}\\ 0 \end{array}\right),n_{TM}^{sph}=\left(\begin{array}{c} \frac{\alpha \gamma^{\prime }}{\sqrt{\alpha^{2}+\beta^{2}}}\\ \frac{\beta \gamma^{\prime }}{\sqrt{\alpha^{2}+\beta^{2}}}\\ \sqrt{1-\gamma^{\prime 2}} \end{array}\right), \end{array}$$

where

$$\begin{array}{ccc} \gamma^{\prime } & = & \cos\left(\theta^{\prime }\right)=\sqrt{1-{\left(\alpha /M\right)}^{2}-{\left(\beta /M\right)}^{2}}=\sqrt{1-{\left(\frac{f}{f^{\prime }}\right)}^{2}\left(\alpha^{2}+\beta^{2}\right)}, \end{array}$$

and (α,β) are the exit pupil coordinates for the microscope objective, the object point is (x,0,δz), *M* is the microscope magnification, *f* is the focal length of the objective, and $f^{\prime }$ is the focal length of the tube lens. We express the Debye/angular spectrum integral for image formation in Cartesian coordinates as an integral over the exit pupil coordinate for the microscope objective

(16) $$\begin{array}{c} E_{img}\left(x^{\prime },y^{\prime };x,y_{0},\delta z\right)=\frac{i}{\lambda }\underset{\sqrt{\alpha^{2}+\beta^{2}}\leq NA}{\int \int }\frac{E_{s}\left(\alpha ,\beta ,\gamma^{\prime },x,y_{0},\delta z\right)}{\gamma^{\prime }}\times \\ e^{-i\left(\left(\alpha /M\right)x^{\prime }+\left(\beta /M\right)y^{\prime }\right)}d\alpha d\beta . \end{array}$$

The Debye integral in Equation (16) is for a confocal microscope with an infinitely small pinhole in the detector plane. In other words, the detector point is $\left(x^{\prime },y^{\prime }\right)=\left(0,0\right)$ resulting in

(17) $$\begin{array}{c} E_{img}\left(0,0;x,y_{0},\delta z\right)=\frac{i}{\lambda }\underset{\sqrt{\alpha^{2}+\beta^{2}}\leq NA}{\int \int }\frac{E_{s}\left(\alpha ,\beta ,\gamma^{\prime },x,y_{0},\delta z\right)}{\gamma^{\prime }}d\alpha d\beta . \end{array}$$

Equation (17) expresses the image field for one $\left(x,y_{0},\delta z\right)$ position of the object. We simulate multiple object and defocus positions by applying Equations (11) and (12). The lateral magnification, *M*, of the system gives us the image position for an object movement of (x,y) through the relation $\left(x^{\prime },y^{\prime }\right)=M\left(x,y\right)$.

The field passing through an infinitely small pinhole for light–matter interaction position $\left(x,y_{0},\delta z\right)$ is a coherent sum of plane-wave components coming from each of the wave vector points (α,β) in the exit pupil of the objective

(18) $$\begin{array}{c} dE_{img}\left(x^{\prime },y^{\prime },z_{img};x,y,\delta z\right)=\frac{i}{\lambda }\frac{E_{s}\left(\alpha ,\beta ,\gamma^{\prime },x,y_{0},\delta z\right)}{\gamma^{\prime }}d\alpha d\beta . \end{array}$$

Equation (18) enables the calculation of the image stack of 2D images found by lateral and vertical scanning of the focal position. A 3D image is obtained from the image stack using the methods in Ref. [21].

### 4.2. FEM Method

As the FEM model used in the simulations shown in this study has been described in detail in previous publications, we do not go into detail here. The FEM modeling of the light–surface interaction is mainly reported in Ref. [22], where FEM is used to simulate results obtained by interference microscopy. In a subsequent publication [8], FEM is combined with confocal microscopy and validated by comparison to measurement results. The simulation used in this study follows this publication with slight modifications in the instrument modeling reported elsewhere [23]. This publication further demonstrates how to develop digital twins for other common 3D microscopy techniques, namely coherence scanning interferometry and focus variation microscopy based on the same theoretical foundations.

### 4.3. BEM Method

The BEM solves Maxwell’s equations through integral formulations and Green’s functions. In contrast to differential equation solvers like FEM, which discretize fields volumetrically across the entire domain, BEM focuses solely on surface discretization, resulting in a smaller number of unknowns. BEM can be significantly more efficient than other methods for certain 3D problems. However, its applicability is limited to homogeneous or piecewise homogeneous domains. Additionally, as the size of the problem increases, the computational expense of BEM also escalates. The fast multipole method and its multilevel version, the multilevel fast multipole method, have thus been developed to speed it up while maintaining a high accuracy. In this research, a simulation tool based on the BEM method accelerated through multilevel fast multipole method for aperiodic 3D structures was utilized to simulate the RS-N grating structures for comparison [24]. An essential difference between BEM and the other two methods, FMM and FEM applied for this research, is that BEM is for modeling 3D isolated structures, whereas the presented FMM and FEM are typically employed for analyzing 2D and 3D periodic structures. Thus, the grating calculated using BEM has a limited size in both the *x*- and *y*-directions, which is an aperiodic 3D object with open boundaries. To simulate a periodic grating, only several periodicities can be included along the *x*-direction, which makes the structure quasi-periodic. Nevertheless, the advantage of the method is that it is feasible to simulate a tilted grating.

Similar to the FEM model, the Fourier optics modeling of the confocal microscopy in the case of BEM follows the method presented in Ref. [8].

## 5. Results and Discussion

In order to investigate the ability of the digital twins to reproduce the true surface of an object, we perform far-field and near-field comparisons. All comparisons are performed for a SiMetricS RS-N silicon resolution standard with 1200 and 3000 nm line grating regions [10]. The 3000 nm pitch region has a height of 189 nm, a width of 1500 nm, and a sidewall angle of 90 degrees. The 1200 nm pitch region has a height of 176 nm, a width of 600 nm, and a sidewall angle of 90 degrees. The samples were illuminated with a wavelength of 405 nm using an infinity-corrected objective with an NA of 0.95 giving the same point spread function for all digital twins. In this context, it should be noted that the confocal microscope detects images from the object’s surface in the far-field. Hence, Section 5.1 shows simulations of near fields, whereas all other sections show far-field results.

### 5.1. Near-Field Comparison

**Figure 3:**
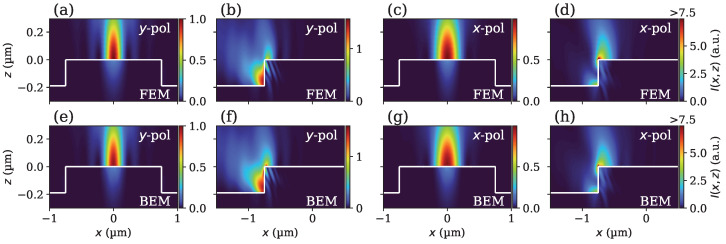
Simulated near-field intensities for confocal illumination with NA = 0.95 and λ=405 nm at the center (**a**,**c**,**e**,**g**) and edge (**b**,**d**,**f**,**h**) of the rectangular RS-N grating. The white lines illustrate the interface between silicon and air. Above this interface is the reflected/scattered field; below this interface, the transmitted field is shown. The light is either y- (**a**,**b**,**e**,**f**) or x- (**c**,**d**,**g**,**h**) polarized. Results are obtained using FEM (**a**–**d**) and BEM (**e**–**h**).

As a first comparison, near fields are obtained for several angles of incidence within a light cone limited by the NA of 0.95 here. In order to simulate confocal illumination, fields for all angles of incidence are superimposed coherently. For more information on the definition of the angles of incidence and implementation of confocal illumination, we refer to previous articles [8,23]. Figure 3 displays intensities simulated by either FEM (Figure 3a–d) or BEM (Figure 3e–h) using y- as well as x-polarized light of λ=405 nm. The finite grating size used for BEM simulation has a length $L_{x}$ of 6 µm along the x-direction, and $L_{y}$ of 2 µm along the y-direction. The confocal illumination is focused on the top center (Figure 3a,c,e,g) or the top-left edge of the grating (Figure 3b,d,f,h). The intensities are all normalized by the maximum intensity obtained from the center for y-polarization (Figure 3a for the top and Figure 3e for the bottom row). Please note that in the case of focusing on the edge with *x*-polarization, the maximum intensity displayed has been adjusted for improved visibility, as singularities with high intensity occur at the edges. Furthermore, only the reflected/scattered field (without the incident field) above the grating is shown. Comparing fields simulated with FEM and BEM results for both polarizations and at the center as well as at the edge of the grating agree well. The field distributions, as well as the magnitude of the intensity, show good agreement for all positions and polarizations. Slight deviations are probably reasoned by the assumption of an infinitely extended periodic profile of FEM compared to a profile limited in the *x* as well as *y* directions considered in the BEM simulation. Nonetheless, these deviations are negligibly small.

### 5.2. Confocal Imaging

The confocal imaging method presented in Section 3 requires a conventional bright-field microscope objective, a tube lens, a pinhole, a point detector such as a photon multiplier tube to capture images, and a motorized stage to move the target through the focus.

**Figure 4:**
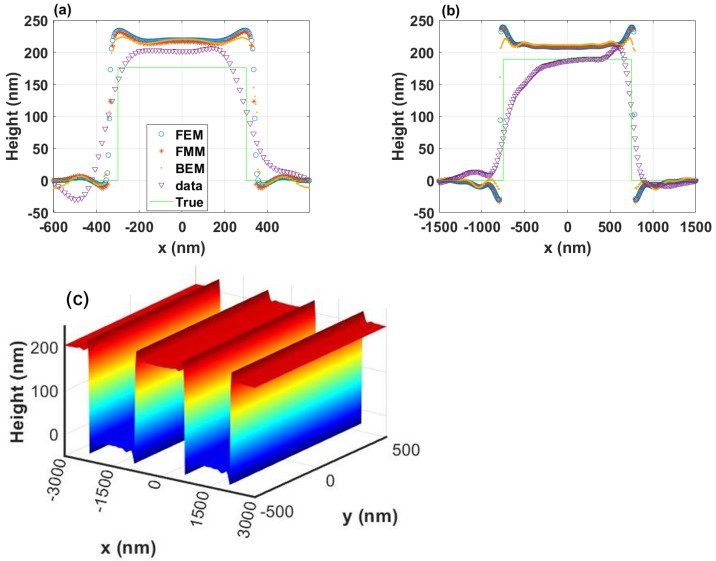
Simulated and experimental optical profile for unpolarized light. Results for (**a**) 1.2 µm pitch and (**b**) 3 µm pitch. (**c**) 3D visualization of grating profile constructed using a 2D profile calculated using FEM shown in (**b**) for 3 µm pitch.

The results in Figure 4 for the digital-twin methods in Section 4 show an excellent agreement between the three methods for unpolarized light in (a) and (b). In Figure 4c, we present a 3D visualization of a section of the grating with $\Lambda_{x}=3000$ nm pitch. The BEM results for the 3000 nm pitched sample show slightly less good agreement with the other two methods, although the near fields from BEM agree well with those from FEM, as shown in Figure 3. This is because of the edge effect with the BEM method. Since the grating calculated using BEM has a limited size in both the x- and y-directions, the light scattered from the edges thus contributes to the light scattered from the structure. To improve the simulation for periodic structures using BEM, we must implement a periodic boundary condition to it and additionally, a periodic Green’s function and the multilevel fast multipole method for periodic structures.

The simulations also show good agreement with the experimental measurement and nominal shape even though all the simulated heights are larger than both the experimental and the nominal heights. The measured optical profile for the 3 μm pitch grating has an asymmetry shape that does not appear in the measured profile for the 1.2 μm pitch grating. The two gratings are etched into the same silicon substrate, so, besides aberrations, the most likely causes of the asymmetry are due to the manufacturing. As demonstrated by Hagemeier et al. [21], the simulated height overestimation follows from the occurrence of batwings at the edges and is reduced for longer period lengths. Besides this effect, we find that the nonlinearity of the problem does not, in general, allow for a systematic correction factor for the height estimation. However, there are general simulation effects that could influence the height estimation. In particular, we expect slight height differences between the simulated and the measured height values as a result of the use of aplanatic lenses and an infinitely small confocal pinhole in the simulations. A first qualitative study on the influence of apodization and finite pinhole size on the measured rectangular grating profiles is published elsewhere [8].

In a similar way, aberrations, apodization, and a finite pinhole size can be considered in the simulation by appropriate pupil functions. However, a detailed analysis of these effects is out of the scope of this paper. Furthermore, discrepancies between the real object’s surface height function and an ideal rectangular grating profile assumed in the simulations cause additional differences between simulated and measured results. The influence of rounded corners and the tilt of the grating is investigated in more detail in subsequent sections.

Deviations between the conventional and the measured height profile mainly result from the scattering process and the diffraction-limited microscopic imaging and are thus adequately considered in the simulation.

#### 5.2.1. Influence of Corner Roundings on Optical Profile

**Figure 5:**
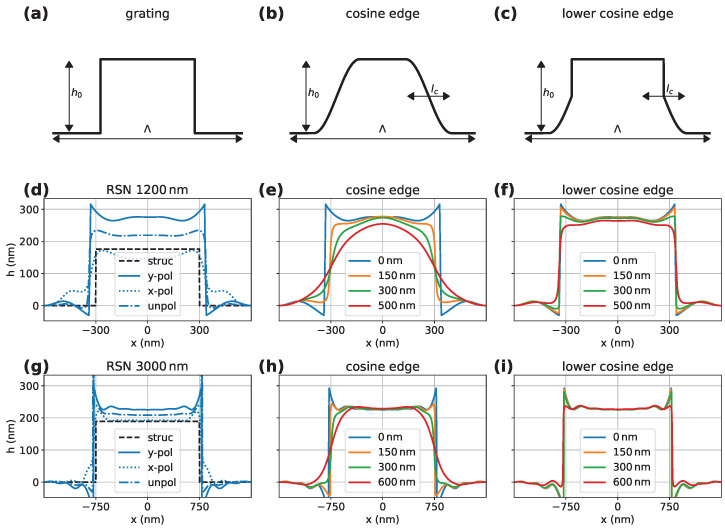
Simulated optical profiles for differently rounded edges; (**a**–**c**) show the different structures, including perpendicular edges (**a**), and rounded edges at the top and bottom corner (**b**), or only at the bottom corner (**c**). (**d**–**i**) show gratings reconstructed from simulated image stacks for different parameter assumptions. In (**d**,**g**) results from a rectangular grating with $\Lambda_{x}=1200$ nm and $\Lambda_{x}=3000$ nm, respectively, are shown for x-, y- and unpolarized light. In the corresponding figures (**e**,**f**) or (**h**,**i**), rounded edges approximated by a half cos function or quarter cos function as shown in (**b**), and (**c**) are considered. Simulation results are presented for both period lengths using y-polarized light.

Previous AFM surface measurements have already shown that the lattice structure of the RS-N deviates from an exact rectangular lattice structure [22]. The edges of the grid structure run at an angle and are almost round at the bottom corner [25]. In order to estimate the influence of the deviation of the real structure from the perfect structures used in the simulations, the edges were replaced by a half or a quarter period of a cosine function. These are shown in Figure 5a–c. The height $h_{0}$, the period length of the grid $\Lambda_{x}$, and half the period length of the cosine $l_{c}$ can be set. Figure 5d,g display the results of the FEM simulation for a perfect grating shown previously in Figure 4. In addition to the results already shown for unpolarized light, the individual x/y components are also shown here. For all three polarizations and both period lengths, the width of the upper plateau is larger compared to the nominal one. This effect is probably attributed to edge diffraction and obscuration occurring at the edges. Furthermore, it can be clearly seen that the *y* component makes the largest contribution to the batwing that occurs. Please note that the observation that the batwing is more pronounced for *y* polarization is not generalizable but depends on the step height and light wavelength as shown on the example of CSI elsewhere [22]. However, for the given setting, the largest batwings occur for y-polarization, which is therefore considered to investigate the behavior of rounded corners in the following.

Figure 5e,h show the results for different degrees of rounding. The strength of the rounding is represented by the length of $l_{c}$. The selected values for $l_{c}$ can be found in the figures with the corresponding colors. The values for $l_{c}=0$ nm correspond to the respective *y* curve from (d) or (g). It is noticeable that even with small roundings that are below the resolution limit, the batwing is already significantly smaller. This may occur due to edge diffraction [26]. It can also be seen that the widths of the upper plateaus are closer to the nominal value, even for small roundings. Consequently, the overestimation of the width can be attributed to edge diffraction effects occurring at sharp edges.

Figure 5f,i also show different degrees of rounding. However, the selected cosine function was only used for the lower edge. It is noticeable that the batwing only drops slightly with increasing rounding. This may be due to the fact that a perfect corner is again assumed for the upper edges. It is also seen that, compared to (e) and (h), the width of the upper plateau is measured too wide even if no batwing is apparent, which is an additional indicator for edge diffraction from the top edge as the reason for this overestimation.

#### 5.2.2. Influence of Sample Tilt on Optical Profile

In order to understand the asymmetry of the measured results shown in Figure 4, the CM signal with a tilted substrate was calculated using BEM. Due to its open boundaries for 3D isolated structures, the method is a good option for such a study.

**Figure 6:**
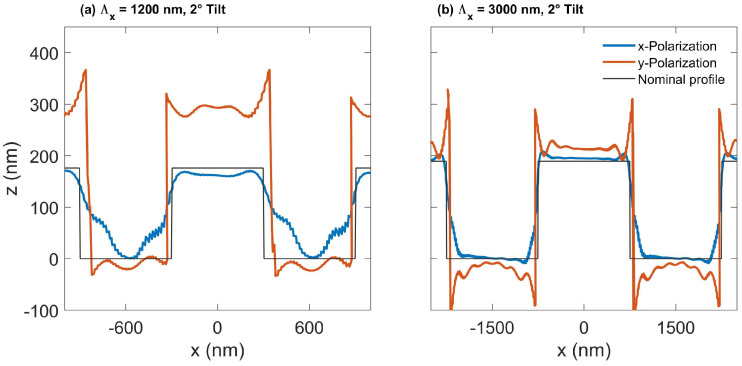
Calculated CM profiles using BEM for gratings with a substrate 2° tilted around the z-axis towards the x-direction, which is the direction perpendicular to the grating. Results from y- and x-polarizations for gratings with (**a**) $\Lambda_{x}=1200$ nm, (**b**) and $\Lambda_{x}=3000$ nm.

Figure 6a shows the results from x- and y- polarizations for the grating $\Lambda_{x}=1200$ nm, and Figure 6b for the grating $\Lambda_{x}=3000$ nm. From both figures, we observe a difference between the x- and y-polarizations, which is caused by the shadowing effect at larger incident angles. This explains why for a larger pitch, such as $\Lambda_{x}=3000$ nm, the difference between the two polarizations is reduced. A substrate tilt, which is 2° in this case, also induces profile asymmetries derived from the CM far-field images. For the grating with $\Lambda_{x}=1200$ nm at *x*-polarization, an obvious asymmetry occurs at the bottom of the grating. At *y*-polarization, the asymmetry is more clearly demonstrated at the top of the grating. For the grating with $\Lambda_{x}=3000$ nm, only a slight asymmetry can be observed for both y- and x-polarizations. We assume that this is for the same reason stated above: with a larger pitch, light from different angles can illuminate the grating more effectively, reducing the shadowing effect. When the substrate is tilted, the shadowing effect is reduced on one side more than on the other. However, we cannot obtain the same results as the measured shown in Figure 4a when we combine the results from the two polarizations together because the illumination might not be a perfect circularly polarized light, aberrations such as asymmetric pupil illumination may exist, and lower order frequencies from the sample are propagated best through the optical system.

### 5.3. Expected Optical Heights for a Range of Gratings

In the development, specification, and use of instruments for measuring areal surface topography, a fundamental need is to characterize the expected response to a given surface geometry. This expected response is called the ITF [27,28,29] and is defined for shallow sinusoidal gratings. In this article, we use FMM to simulate the optical height for twelve rectangular gratings. Nine of these gratings are SiMetricS RS-N calibration gratings with nominal pitches from 300 nm to 6000 nm, nominal heights from 138 nm to 192 nm, and 50% duty cycle [10]. The last three rectangular gratings have pitches of 150 nm, 200 nm, and 250 nm with a nominal height of 138 nm and 50 % duty cycle. All simulations have been performed with a NA of 0.95 and a wavelength of 405 nm.

**Figure 7:**
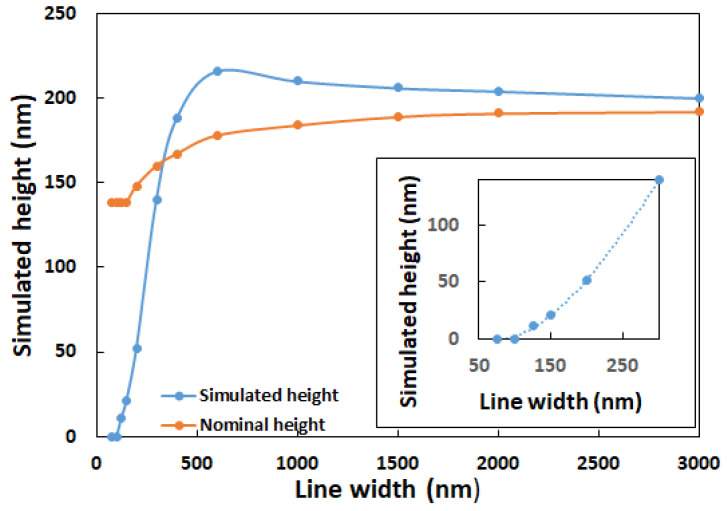
Nominal input heights and the simulated optical heights for unpolarized light as a function of grating line width. The insert shows a close-up of the low line width region.

Figure 7 shows the nominal input heights and the simulated optical heights for unpolarized light as a function of the grating line width. From the figure we observe that the region with line widths between 400 nm and 3000 nm all have heights larger than the nominal height due to the aforementioned batwing effect that falls off towards the center but might still give height contribution. Furthermore, we observe that the batwing effect leads to an overestimation of heights that decreases with larger period lengths. For the region with line widths below 400 nm, we observe that the simulated optical heights are less than the nominal heights, indicating that the microscope objective does not capture all the spatial frequencies from the grating structure and damping due to the transfer characteristics of the system [30,31]. The insert in the figure shows that a grating with 100 nm line width gives a simulated optical height of zero. This limit is called the resolution limit for the optical system.

## 6. Conclusions

This paper presents a comparison of three digital-twin models for confocal microscopy, developed independently by the authors, that investigate the possibility of transforming a conventional confocal microscope into a 3D metrology tool with nanometer sensitivity. Figure 4 shows that it is possible to achieve consistent optical simulation for very different simulation methods and that it is possible to theoretically observe the batwings and to observe that the impact of the batwings is smallest at the center of the ridge. Figure 7 shows that the impact of the batwings continues to increase with decreasing pitch until the pitch becomes so small that the microscope cannot fully resolve the structure.

The influence of corner roundings on optical profiles has also been investigated. We observe that the presence of upper and lower corner roundings reduces the batwings significantly, whereas the batwings are reduced at a much lower rate if we only have lower roundings. The investigation shows that corner roundings may be a candidate for explaining why we see much fewer batwings in experimental measurements. This work furthermore suggests that simulating the manufactured profile, including corner roundings, instead of the designed rectangular profile gives better agreement between experimental and simulated results. The outcome of the influence of sample tilt on the optical profile is less obvious. We do observe asymmetry in the simulated structure, but connecting with the experimental measurement is difficult, indicating that more physical effects may contribute to the observed asymmetry. The expected optical height for a number of rectangular gratings with pitches from 150 nm to 6000 nm is simulated, and we observe that the minimum separation distance that can be measured between two lines is 100 nm. Beyond this resolution limit, we first observe a fast increase in optical height due to capturing more spatial frequencies from the grating structure with increasing pitch. Followed by a slowly varying optical height curve that approaches the nominal height values.

The near-field interaction between the focused light and the object has been investigated in two cases. In the first case, the focal point is on the top of the center ridge, and in the second case, the focal point is on the edge of the ridge. The observed intensity patterns are quite different for the two cases. In the first case, we observe nice symmetrical intensity profiles for both x- and y-polarized incident light. In the second case, we observe strong asymmetries in the intensity profiles for both polarization at the edge. In the case of focusing on the edge with x-polarization, we observe singularities of high intensity at the top corner, whereas the intensity in the y-polarized case is more evenly distributed along the sidewall.

No formal analysis of experimental uncertainty and simulation errors have been included in this article since the Olympus microscope is only traceable for heights of individual lines. Accurate line-width measurements are currently in development and will, in general, require excellent sample quality for the inter-comparison and systematic error control of the optical instruments. The systematic errors may be investigated by comparing the response from a perfect digital-twin simulation and a digital-twin simulation that includes the major error sources, including camera noise, environmental vibration, two-dimensional angular deviations between the measurement optical axis and specimen plane, nonlinearity of the scanner along the optical axis, and optical aberrations.

In summary, this article demonstrates that a confocal microscope can be transformed into a 3D microscope metrology system in the micro/nanometer range. Furthermore, it highlights that the digital twins developed in this article are demonstrated to agree well under the same circumstances, although they were developed at different institutes and initialized by varying setups. This represents a great step forward in the simulation of optical surface topography measurement instruments.

## Data Availability

The corresponding author will respond to reasonable requests for details in the implemented methods.

## Abbreviations

The following abbreviations are used in this manuscript:

AFM	Atomic force microscopy
BEM	Boundary element method
BFP	Back focal plane
CSI	Coherence scanning interferometry
FDTD	Finite difference time domain
FEM	Finite element method
FMM	Fourier modal method
ITF	Instrument transfer function
LED	Light-emitting diode
NA	Numerical aperture
OM	Optical microscopy
RCWA	Rigorous coupled-wave analysis
SEM	Scanning electron microscopy
TE	Transverse electric
TM	Transverse magnetic
2D	Two-dimensional
3D	Three-dimensional
